# Supplementary material for: Cloud BioLinux: pre-configured and on-demand bioinformatics computing for the genomics community
Source: BMC Bioinformatics. 2012 Mar 19;13:42. doi: 10.1186/1471-2105-13-42 (PMC3372431; doi:10.1186/1471-2105-13-42)
Supplement: Additional file 1 — Supplementary 1 Cloud BioLinux software documentation in the form of a mini, self-contained website. Users need to download and uncompress the .zip file, and open through a web browser the "index.html" file available on the main directory. (ZIP 1823 kb). [file 1471-2105-13-42-S1.ZIP › Cloud-BioLinux-Package-Documentation/Pipelines.html]

Bio-Linux Software Documentation Pages

Back to search form

### Pipelines

|  |  |
| --- | --- |
| Category | Applications |
| Pipelines | Taverna    run-mummer3    nucmer |
| SubCategory | Applications |
| 16S processing | qiime |
| Sequence\_assembly | gap4    partigene    stars |
| Sequence\_processing | nucmer    pregap4    run-mummer3    stars    trace2dbest |
| Sequence\_typing | stars |
| Workflows | Taverna |
